# The effect of anesthetic dose on the motor response induced by low-intensity pulsed ultrasound stimulation

**DOI:** 10.1186/s12868-018-0476-2

**Published:** 2018-12-03

**Authors:** Yi Yuan, Xingran Wang, Jiaqing Yan, Xiaoli Li

**Affiliations:** 1 Institute of Electrical Engineering, Yanshan University, Qinhuangdao, 066004 China; 2 College of Electrical and Control Engineering, North China University of Technology, Beijing, 10041 China; 3 State Key Laboratory of Cognitive Neuroscience and Learning, Beijing Normal University, Beijing, 100875 China

**Keywords:** LIPUS, Anesthetic dose, Mouse, Motion response

## Abstract

**Background:**

Low-intensity pulsed ultrasound stimulation (LIPUS) has been proven to be a noninvasive method with high spatial resolution and deep penetration. Previous studies have qualitatively demonstrated that the electromyographic response caused by LIPUS in the mouse motor cortex is affected by the anesthetic state of the mice. However, the quantitative relationship between motor response and anesthetic dose remains unclear.

**Results:**

Experimental results show that the success rate decreases stepwise as the isoflurane concentration/mouse weight ratio increases (ratios: [0.004%/g, 0.01%/g], success rate: ~ 90%; [0.012%/g, 0.014%/g], ~ 40%; [0.016%/g, 0.018%/g], ~ 7%; 0.024%/g, 0). The latency and duration of EMG increase significantly when the ratio is more than 0.016%/g. Compared with that at ratios from 0.004 to 0.016%/g, normalized EMG amplitude decreases significantly at ratios of 0.018%/g and 0.020%/g.

**Conclusions:**

Quantitative calculations indicate that the anesthetic dose has a significant regulatory effect on the motor response of mice during LIPUS. Our results have guiding significance for the selection of the anesthetic dose for LIPUS in mouse motor cortex experiments.

## Background

Low-intensity pulsed ultrasound stimulation (LIPUS) has been proven to be a noninvasive method with high spatial resolution and a deep penetration depth [1–3]. The potential mechanism of ultrasound stimulation is that the ultrasound alters membrane conductance or membrane capacitance to induce a current effect [4, 5]. LIPUS modulates (excites or inhibits) neuronal activity and causes neural oscillations, which not only reflect the characteristics of the brain activity itself but also yield clues into the underlying associated neural dynamics [6, 7]. Evidences shows that LIPUS can induce neural responses in vitro [8], promote protein expression [9, 10], induce a hemodynamic response [11, 12], and causally modulate brain activity [13–15], including the induction of motor responses [16, 17].

Previous studies demonstrated that the motor response induced by LIPUS is related to the state of anesthesia. Kim et al. [18] used low-intensity ultrasound to successfully stimulate the brain motor area in Sprague–Dawley rats with light anesthesia to examine the range of sonication parameters that minimize acoustic intensity/energy deposition. Mehić et al. [19] stimulated lightly anesthetized mice by using a transcranial modulated-focus ultrasound and produced various motor movements with high spatial selectivity to increase the anatomical specificity of neuromodulation. King et al. [20] used ultrasound to stimulate the mice somatomotor cortex and recorded the electromyography (EMG) signal to evaluate the somatomotor response. Their results showed that the stimulation success rates were 10%, 98.7%, and 94.6% when the mice had anesthesia levels of 0.5%, 0.1%, and 0.02% isoflurane. They found that ultrasound-evoked contractions were rare at 0.5% isoflurane but became more frequent as the anesthesia level decreased. Younan et al. performed a study in which mice under light and deep anesthesia were stimulated by ultrasound. They found that there were different motor responses between light and deep anesthesia [21]. The abovementioned studies demonstrated that the strength of the anesthesia is relevant to inducing motor responses by LIPUS in rodents. However, the quantitative relationship between the motor response and the anesthetic dose is still unclear.

In this study, we used isoflurane at different concentrations to anaesthetize mice with weights of 25 ± 0.5 g. The ratios of isoflurane concentration to mouse body weight ranged from 0.004 to 0.024%/g with an interval of 0.002%/g. Low-intensity pulsed ultrasound was used to stimulate the mouse motor cortex after 10 min of anesthesia. Simultaneously, the EMG data from the tail were recorded. The success rate of the motion response and the latency, duration and amplitude of the EMG signal were analyzed.

## Methods

### Animal anesthesia and surgery

We used eleven BALB/c mice for the experiments (all male, body weights ~ 25 g, Beijing Vital River Laboratory Animal Technology Co., Ltd. China). Our study protocols were submitted to and approved by the Animal Ethics and Administrative Council of Yanshan University (No. S201700135). 2% isoflurane (RWD Life Science Co. Shenzhen, China) was used for surgical anesthesia in the experiment. The anesthetized mice were fixed in a stereotaxic apparatus (ST-5ND-C, Stoelting Co., USA) with ear bars and a clamping device. We shaved the fur covering the animal’s skull and cleaned the skin with a physiological 0.9% sodium chloride solution. The mice were sacrificed with an overdose of anesthetic (25% isoflurane) when the experiment was finished.

### LIPUS experimental setup

**Fig. 1:**
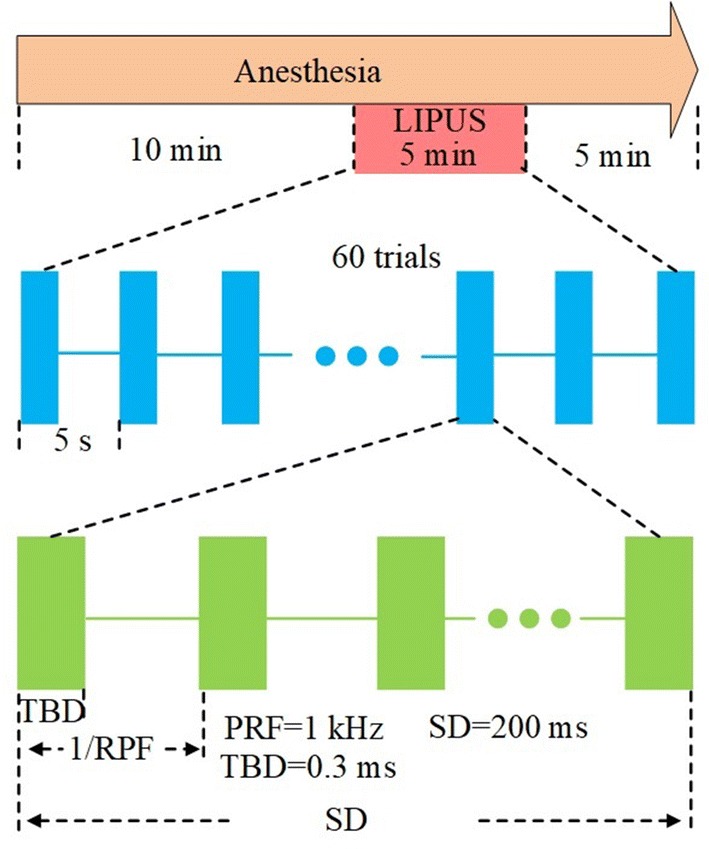
The sequence diagram of the LIPUS and ultrasound parameters

The LIPUS system was similar to that used in our previous paper [22]. An unfocused ultrasound transducer (V301-SU, Olympus, USA) with FF of 500 kHz was used to generate ultrasound wave. A conical collimator filled with ultrasound coupling gel was used to connect the transducer and mouse skull. The sequence diagram of the ultrasound stimulation is shown in Fig. 1. The PRF, SD and TBD of the ultrasound were 1 kHz, 200 ms and 0.3 ms, respectively. The ultrasound pressure was measured by a calibrated needle-type hydrophone (HNR500, Onda, Sunnyvale, CA) and the corresponding spatial-peak and pulse-average intensity (I_sppa_), was ~ 2 W/cm^2^.

### Data acquisition

An EMG electrode was attached to the tail of each mouse, and an EMG common ground wire was inserted into the back of each mouse. The EMG signals was collected by a 32-channel neural signal processor (Cerebus Data Acquisition System, Blackrock Microsystems, USA). The raw EMG signals produced in response to LIPUS were acquired at a sampling frequency of 2 kHz in 4 s trial epochs.

### Experimental process

In the experiment, the isoflurane was used to anesthetize the mice. The anesthetic doses of isoflurane that were chosen in LIPUS were 0.1%, 0.15%, 0.2%, 0.25%, 0.3%, 0.35%, 0.4%, 0.45%, 0.5%, 0.55%, and 0.6%, respectively. Since the mice had body weights of ~ 25 g, the corresponding ratios of isoflurane concentration to mouse body weight were 0.004%/g, 0.006%/g, 0.008%/g, 0.01%/g, 0.012%/g, 0.014%/g, 0.016%/g, 0.018%/g, 0.020%/g, 0.022%/g and 0.024%/g, respectively. First, the mice were anesthetized with one concentration for 10 min before LIPUS. Next, LIPUS was performed for 5 min. At the same time, the EMG signal from the tail was recorded. Last, the anesthesia was continued for 5 min. When the experiment was finished, an experiment with another anesthetic dose was performed.

### Statistical analysis

Data are presented in the form of means ± standard errors of the means (S.D.). The primary statistical analysis used in the present study was the paired sample t-test. When the p-value is less than 0.05, the results were considered to be statistically significant.

## Results

**Fig. 2:**
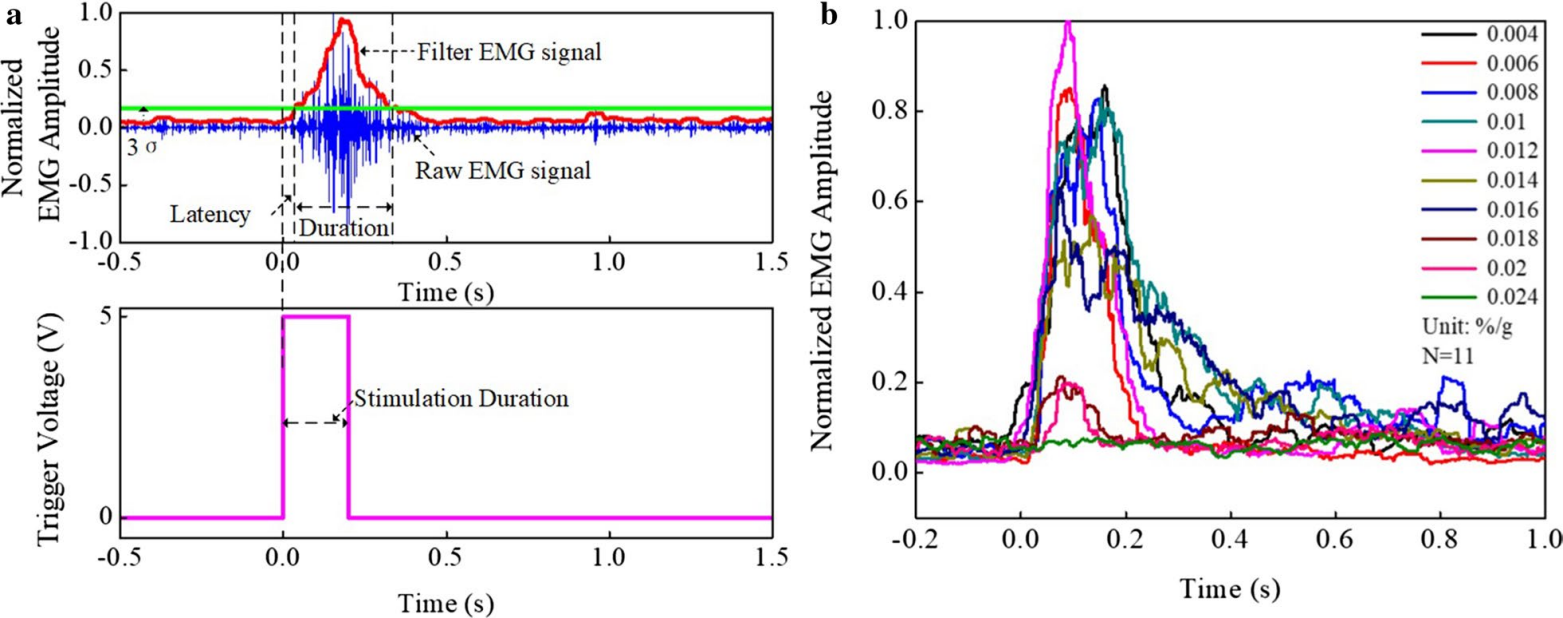
**a** A sample EMG signal from one mouse (above) and a trigger signal marked the ultrasound emission (below). **b** The rectified, smoothed EMG signals corresponding to ten different ratios of isoflurane concentrations and the mouse body weight selected from 0.004 to 0.024%/g at an equal interval of 0.002%/g, which is represented by different color lines (N = 11)

The upper image of Fig. 2a illustrates a sample EMG signal from one mouse, and the lower image of Fig. 2a shows the trigger signal marking the ultrasound emission. We can see that there is an obverse EMG signal after LIPUS. As shown in Fig. 2b, the smooth EMG curves that correspond to the ratios of isoflurane concentrations and mouse body weights have similar trends of change. It can be seen that the curves corresponding to the ratios of 0.004–0.016%/g do not have obvious differences. Nevertheless, the amplitude from 0.018 to 0.024%/g decreased significantly.

**Fig. 3:**
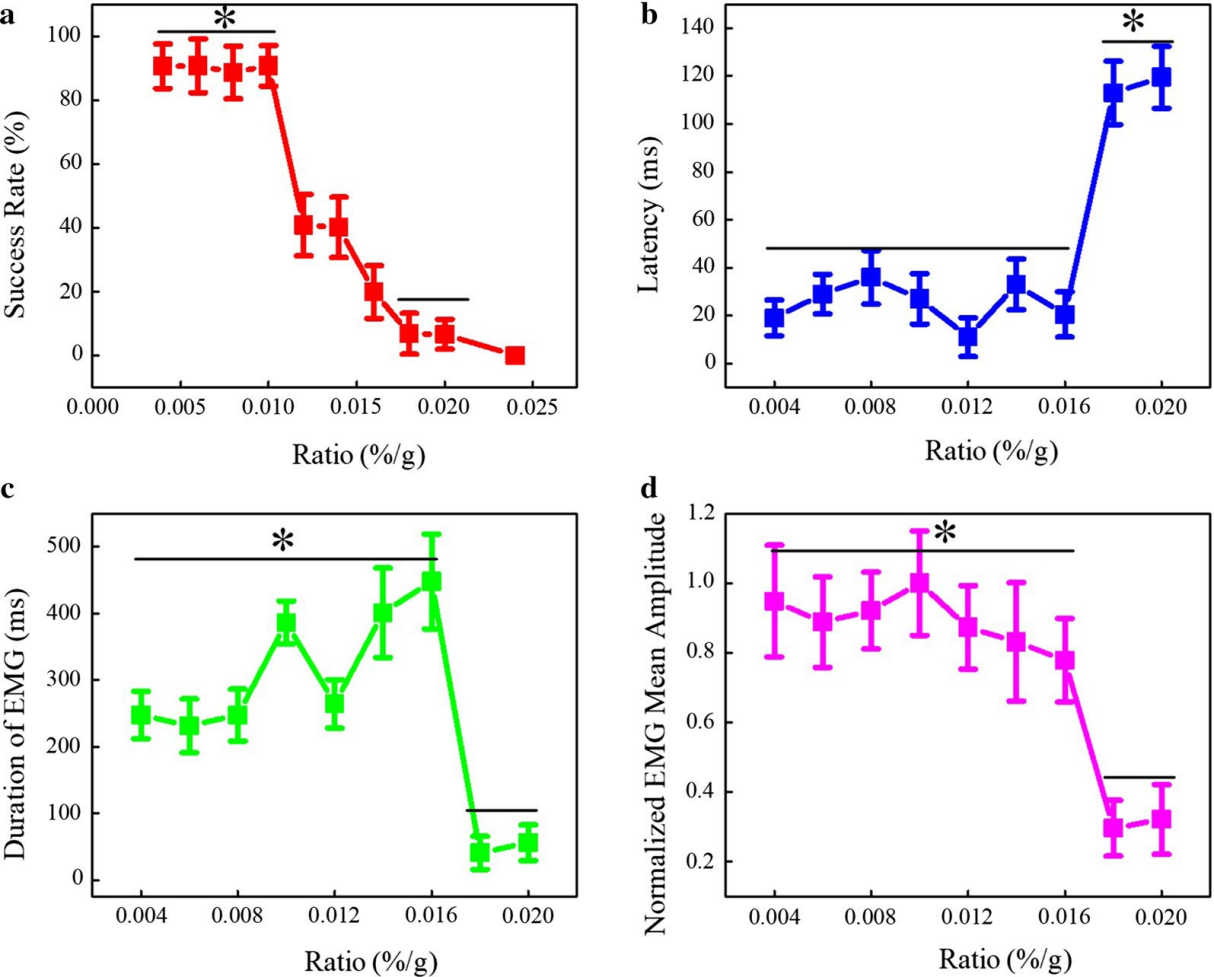
**a** The success rates of motor response: 90.6 ± 6.9%, 90.7 ± 8.4, 88.6 ± 8.2, 90.7 ± 6.3, 40.8 ± 9.6, 40.1 ± 9.4, 19.9 ± 8.2, 6.8 ± 6.3, 6.6 ± 4.6 and 0 with different ratios from 0.004 to 0.024%/g (N = 11, mean ± S.D., paired t-test, *p < 0.05, df = 10, t values shown in Table 1). **b** The latency of the EMG signal. (0.004%/g: 19.1 ± 7.5 ms, 0.006%/g: 29.2 ± 8.3 ms, 0.008%/g: 36.1 ± 11.2 ms, 0.01%/g :27.5 ± 10.5 ms, 0.012%/g:11.3 ± 8.1 ms, 0.014%/g: 33.2 ± 10.6 ms, 0.016%/g: 20.5 ± 9.5 ms, 0.018%/g: 113.2 ± 13.3 ms, 0.020%/g: 119.5 ± 12.9 ms) (N = 11, mean ± S.D., paired t-test, *p < 0.05, df = 10, t values shown in Table 1). **c** The duration of EMG (0.004%/g: 247.5 ± 35.5 ms, 0.006%/g: 231.5 ± 40.2 ms, 0.008%/g: 247.5 ± 38.9 ms, 0.01%/g: 386.5 ± 32.2 ms, 0.012%/g: 264.5 ± 36.3 ms, 0.014%/g: 401.2 ± 67.2 ms, 0.016%/g: 448.3 ± 71.1 ms 0.018%/g:40.8 ± 25.1 ms, 0.020%/g: 56 ± 26.7 ms) (N = 11, mean ± S.D., paired t-test, *p < 0.05, df = 10, t values shown in Table 1). **d** The normalized amplitude of the EMG signal with different ratios. (N = 11, mean ± S.D., paired t-test, *p < 0.05, df = 10, t values shown in Table 1)

To quantitatively evaluate the effect of the anesthetic dose on the motor response induced by LIPUS, the success rate of the motor response, latency, duration and normalized amplitude of the EMG signal were calculated from the upper image of Fig. 2a using the methods detailed in [20]. We referred to the King et al. reference to define the success rate, which is the ratio of the number of contractions identified by using these rules divided by the total number of sonication cycles attempted, expressed as a percentage. As shown in Fig. 3a, the success rates of the motor response were 90.6 ± 6.9%, 90.7 ± 8.4, 88.6 ± 8.2, 90.7 ± 6.3, 40.8 ± 9.6, 40.1 ± 9.4, 19.9 ± 8.2, 6.8 ± 6.3, 6.6 ± 4.6 and 0 with different ratios from 0.004 to 0.024%/g (N = 11, mean ± S.D., paired t-test, *p < 0.05, df = 10, t values shown in Table 1). The results indicate that the success rate decreases stepwise as the ratio of isoflurane concentration to mouse body weight increases. Latency plays an important role in the timing control of EMG [23], and its changes are closely related to neuromuscular control [24]. The duration is related to muscle retardation [25]. Therefore, both of them are used to analyze the motor response. Figure 3b shows the latency of the EMG signal. We can see that the latency was 29.2 ± 8.3 ms, 36.1 ± 11.2 ms, 27.5 ± 10.5 ms, 11.3 ± 8.1 ms, 33.2 ± 10.6 ms, and 20.5 ± 9.5 ms as the ratio increased from 0.004 to 0.016%/g. Then, as the ratio continued to increase to 0.020%/g, the delay time value increased rapidly and finally stabilized at approximately 120 ms (0.018%/g: 113.2 ± 13.3 ms, 0.020%/g: 119.5 ± 12.9 ms) (N = 11, mean ± S.D., paired t-test, *p < 0.05, df = 10, t values shown in Table 1). As shown in Fig. 3c, the duration of EMG slowly rises to nearly 500 ms in fluctuation when the ratio increased from 0.004 to 0.016%/g (0.004%/g: 247.5 ± 35.5 ms, 0.006%/g: 231.5 ± 40.2 ms, 0.008%/g: 247.5 ± 38.9 ms, 0.01%/g: 386.5 ± 32.2 ms, 0.012%/g: 264.5 ± 36.3 ms, 0.014%/g: 401.2 ± 67.2 ms, 0.016%/g: 448.3 ± 71.1 ms). When the ratio increased to 0.018%/g, the duration of EMG decreased rapidly and finally stabilized between 0 and 100 ms (0.018%/g: 40.8 ± 25.1 ms, 0.020%/g: 56 ± 26.7 ms). (N = 11, mean ± S.D., paired t-test, *p < 0.05, df = 10, t values shown in Table 1). Figure 3d shows the normalized amplitude of the EMG signal with different ratios. We can see that the ratio increased from 0.004 to 0.016%/g. Meanwhile, the normalized EMG mean amplitude reduced from 1.0 to 0.8 in fluctuation. Then, with the ratio increasing to 0.018%/g, the normalized EMG mean amplitude value dropped quickly to below 0.4 and finally resided between 0.2 and 0.4. (N = 11, mean ± S.D., paired t-test, *p < 0.05, df = 10, t values shown in Table 1). The results indicate that the anesthetic dose significantly influences the success rate and the EMG latency, duration and normalized amplitude induced by LIPUS. There is a step change in the motor response, especially when the ratio of the anesthetic dose and the body weight is more than 0.016%/g.

**Table 1:** Statistical results of t values

t values	Success rate		Latency		Duration		Amplitude	
	0.018%/g	0.020%/g	0.018%/g	0.020%/g	0.018%/g	0.020%/g	0.018%/g	0.020%/g
0.004%/g	25.7	33.4	− 20.1	− 24.7	15.6	14.9	11.4	11.9
0.006%/g	20.6	34.2	− 18.9	− 19.9	11.1	10.7	12.4	10.6
0.008%/g	33.7	28.2	− 16.3	− 16.3	19.5	19.2	17.6	22.9
0.010%/g	24.5	42.1	− 20.5	− 20.5	17.1	16.3	14.9	13.5
0.012%/g			− 23.5	− 23.5	17.9	15.5	16.5	14
0.014%/g			− 27.7	− 27.7	16.6	15.3	10.9	10.1
0.016%/g			− 23.8	− 23.8	15.9	14.9	8.8	7.9

## Discussions

We designed and performed this study to investigate the effect of anesthetic dose on the motor response induced by LIPUS. By changing the ratio of isoflurane concentration to mouse body weight, we were able to observe obvious variations in muscle contraction and significant changes in the success rate and the latency, duration and the normalized amplitude of the EMG signals in the tail. The findings provide good evidence for the effect of anesthetic dose on the mouse motor response induced by LIPUS.

When the anesthesia was set at a low concentration (ratio of isoflurane concentrations over mouse body weight < 0.016%/g), we could induce tail movement in response to the ultrasound stimulation. Our work confirmed the existence of an anesthetic threshold for motor stimulation with a low-intensity ultrasound. We noticed that there were no motor responses when the ratio reached approximately 0.024%/g. The experimental results were consistent for the whole experimental session and always demonstrated an anesthetic threshold, as shown in Fig. 3a–d. We confirmed that the excitability of the motor cortex was highly dependent on the anesthetic dose.

LIPUS provides a promising new approach for the noninvasive modulation of brain activity and has numerous potential applications in the treatment of neurologic and psychiatric disease, such as epilepsy [26], stroke [27], depression [28] and disorders of consciousness [29]. In our study, we found that the anesthetic dose can affect the neuromodulation effect of ultrasound on the motor cortex when we used the ultrasound to stimulate different rodent disease models under anesthesia. The anesthetic dose may also have an effect on the modulation effect. In our next study, we will further investigate the therapeutic effect of a low-intensity ultrasound on the rodent disease model at different anesthetic doses.

It is very important to ensure safety during LIPUS because ultrasound can induce thermal effects in tissue. The potential temperature increase due to ultrasound parameters can be estimated by the equation $\Delta T = \frac{{2\alpha I{\text{t}}}}{{\rho_{b} C_{p} }}$ [30], where *α* is the absorption coefficient and equals 0.0175 cm^−1^; *I* is the ultrasonic intensity; *t* is the pulse duration of ultrasound; *ρ*_*b*_ is the density of brain tissue; *C*_*p*_ is the specific heat of brain tissue; and the product *ρ*_*b*_ *C*_*p*_ is equal to 3.811 J cm^−3^ °C^−1^. In our study, the maximum ultrasonic intensity was *I* = 2 W/cm^2^, and the pulse duration was *t* = 0.2 s. Therefore, the maximum temperature enhancement induced by LIPUS would be ~ 3.67 × 10^−3^ °C, which is far below the temperature threshold predicted to induce tangible thermal bioeffects.

## Conclusions

By comparing the changes in the success rate and in the latency, duration and normalized amplitude of the EMG signal with increasing ratios of isoflurane concentration to mouse body weight, it can be determined that the anesthetic dose has a significant regulatory effect on the motor response of mice. For ultrasound stimulation in mouse experiments, a good success rate can be obtained when the ratio selected as an anesthetic dose is less than 0.016%/g. The aforementioned results have guiding significance for the selection of the dose of animal anesthesia during LIPUS.

## Abbreviations

LIPUS: low-intensity pulsed ultrasound stimulation
EMG: electromyography
PRF: pulsed repetition frequency
SD: stimulation duration
FF: fundamental frequency
TBD: tone-burst duration
AI: acoustic intensity
I_sppa_: spatial-peak and pulse-average intensity
